# Association of *Helicobacter pylori* with *Candida albicans* enhances fungal virulence and stress tolerance

**DOI:** 10.1186/s13099-026-00831-7

**Published:** 2026-04-09

**Authors:** Jianchao Sun, Qing Luo, Tingxiu Yang, Xiaoli Xu, Tingting Luo, Huifeng Jian, Xianli Chen, Guzhen Cui, Zhenghong Chen

**Affiliations:** 1 Guizhou Key Laboratory of Microbio and Infectious Disease Prevention & Control, School of Basic Medical Science, Guizhou Medical University, Guiyang, China; 2 Key Laboratory of Endemic and Ethnic Diseases of the Ministry of Education & Key Laboratory of Environmental Pollution Monitoring and Disease Control of the Ministry of Education, Guizhou Medical University, Guiyang, China; 3 Guizhou Provincial Center for Clinical Laboratory, Guiyang, China; 4 The Affiliated Cancer Hospital of Guizhou Medical University, Guiyang, China; 5 Department of Hospital Infection and Management, Guizhou Province People’s Hospital, Guiyang, China

**Keywords:** *Candida albicans*, *Helicobacter pylori*, microbial interaction, virulence, drug tolerance

## Abstract

**Background:**

Bacterial associations with fungal hosts are increasingly recognized as common rather than exceptional. Among these interactions, Candida spp. and Helicobacter pylori are of particular interest, as evidence suggests that Candida may serve as a potential host for H. pylori, facilitating its persistence and dissemination. Although interactions between Candida spp. and members of the bacterial microbiota—particularly H. pylori—are increasingly recognized for their role in modulating microbial ecology and influencing pathological outcomes in the host, the impact of H. pylori on Candida albicans remains poorly characterized. Whether this interaction alters the biological behavior or pathogenic potential of Candida spp. remains poorly understood. In this study, C. albicans strains CacoHp, which were detected to be positive for H. pylori–specific genes, were generated through co-culture, and the effects of this bacterial–fungal interaction on tolerance and virulence were investigated.

**Results:**

Co-culture with H. pylori S7 and C. albicans SC5314 yielded Hp-positive C. albicans CacoHp. Compared with the parental strain SC5314, CacoHp exhibited increased tolerance to antifungal agents, sodium dodecyl sulfate, and H_2_O_2_; enhanced inhibition of GES-1 cell proliferation; elevated aspartic protease secretion; and increased hyphal formation. Proteomic and quantitative polymerase chain reaction analyses indicated upregulation of ergosterol transport (SNQ2) and vacuolar ATPase (VMA8) pathways, and enhanced stress tolerance. In mice, CacoHp induced a stronger inflammatory response and more severe gastric tissue damage than the SC5314 strain.

**Conclusions:**

Co-culture with H. pylori generates C. albicans strains CacoHp, which were detected to be positive for H. pylori–specific genes, that exhibit enhanced chemical stress tolerance and increased virulence. This study first revealed the influence of H. pylori on Candida phenotypes, further supporting the clinical relevance of their interaction.

## Background

*Candida* species are among the most prevalent commensal fungi in the human microbiome and coexist with hundreds of bacterial species [1]. *Candida albicans* functions not only as a constituent of the normal microbial flora but also as a significant opportunistic pathogen. Under specific physiological or immunological conditions, these species can cause infections ranging from mucocutaneous candidiasis to life-threatening systemic disease. In recent years, *C. albicans* has emerged as the fourth leading cause of nosocomial bloodstream infections and is frequently isolated from immunocompromised patients [2]. Consequently, the incidence of both localized and systemic opportunistic *Candida* infections has increased, accounting for approximately 8%–10% of hospital-acquired bloodstream infections [3]. Longitudinal molecular typing studies have demonstrated that the majority of *Candida* infections originate from commensal strains rather than from horizontal or vertical transmission [4]. Critically, in many contexts, *Candida* undergoes a phenotypic shift from a benign commensal to an invasive pathogen [5]. The primary virulence mechanisms underlying this transition include adherence to various surfaces, morphological switching (particularly the yeast-to-hypha transition), phenotypic and genotypic plasticity, and the production of lytic enzymes [6].

The pathogenic characteristics of *C. albicans* are associated with multiple factors, including aspartic proteinase activity, drug tolerance, and the ability to withstand environmental stress. *Candida* virulence factors are regulated by multiple genes. Upon internalization into fungal cells, bacteria are capable of regulating the gene expression of the host via multiple signaling mechanisms [7]. The diffusible small molecules secreted by bacteria play a pivotal role in cross-kingdom regulation. For example, quorum-sensing molecules (such as acylated homoserine lactones) may be detected by fungi; notable research has revealed that bacteria can directly perceive fungal quorum-sensing signals like tyrosol to modulate their own antifungal responses [8]. Volatile organic compounds (VOCs) can mediate long-distance signal transduction in a gaseous form and have been shown to significantly alter fungal morphology and physiology [9]. Furthermore, secondary metabolites (including certain antibiotics) can directly inhibit or stimulate specific fungal gene expression networks [10]. These chemical signals, in conjunction with physical internalization processes, collectively constitute the complex mechanism through which bacteria precisely regulate the physiological and transcriptional states of their fungal hosts. Among the numerous virulence-associated genes in *C. albicans*, the role of *C. albicans END3* in efficient endocytosis is indispensable for maintaining cell wall integrity, facilitating protein secretion, promoting hyphal formation, and regulating virulence-related processes [11]. *ENO1* is essential for *C. albicans* growth and contributes to virulence and hyphal development [12]. *SNQ2* facilitates the transport of ergosterol to the cell membrane, maintaining optimal levels of this essential lipid and mitigating damage caused by external stressors such as azoles and other antibiotics [13]. *VMA8* encodes a vesicular proton-transporting ATPase whose adenosine triphosphate (ATP) hydrolytic activity generates a proton gradient that drives the accumulation of ions, amino acids, and metabolites into the vacuole [14]. Alterations in the expression of these genes can significantly affect the virulence and stress tolerance of *C. albicans*.

*Helicobacter pylori* is a Gram-negative pathogen that colonizes the human stomach, with a global infection rate of approximately 50% [15]. It is a primary cause of gastritis and peptic ulcer disease, and 1%–10% of infected individuals may progress from chronic active gastritis to gastric mucosal atrophy, intestinal metaplasia, and ultimately gastric cancer or mucosa-associated lymphoid tissue lymphoma [16]. *H. pylori* infection represents the principal risk factor for non-cardia gastric cancer, as chronic gastric mucosal infection leads to progressive atrophic gastritis and intestinal epithelial hyperplasia [17–19]. Most infected individuals remain asymptomatic, and targeted eradication therapy significantly reduces the risk of malignant transformation.

As early as the 1980s, it was reported that, in addition to *H. pylori*,* Candida* species—particularly *C. albicans*—are capable of colonizing the human gastric mucosa [20]. *C. albicans* has been isolated from gastric mucosal samples of patients with gastric ulcers, gastritis, and duodenal ulcers [21]. In 2005, Siavoshi et al. observed fast-moving, bacteria-like bodies within *Candida* vesicles under light microscopy, which were subsequently confirmed by polymerase chain reaction (PCR) to be *H. pylori* [22]. Since then, multiple techniques have been employed to confirm the presence of *H. pylori* within *Candida*, including detection of *H. pylori*-specific genes and immunoassay-based identification of bacterial proteins in *Candida* species [23–25]. In our previous studies, *H. pylori* 16 S rRNA was detected in vaginal and gastric *Candida* isolates, and *H. pylori*-specific genes and antigens were identified in vaginal and fecal *Candida* samples from neonates and their mothers with *H. pylori* infection [26, 27]. Furthermore, using magnetic beads labeled with *H. pylori*-specific antibodies, *H. pylori* released from *Candida* cells was captured and visualized by scanning electron microscopy [28]. *Candida* functions as a Trojan horse by providing a protective niche that enables *H. pylori t*o evade adverse environmental factors [29]. The internalization of *H. pylori* into *Candida* vacuoles thus represents a potential survival strategy.

Collectively, these findings highlight a potential symbiotic relationship between *Candida* and *H. pylori* under certain conditions. However, research on *Candida–H. pylori* interactions has predominantly focused on the effects on *H. pylori*, often overlooking potential changes in *Candida* during this microbial interplay. Emerging evidence from studies on broader microbial interactions suggests that internalized bacteria can actively modulate fungal gene expression [7]. This regulation may occur through direct mechanisms, such as the delivery of bacterial effector molecules that interact with fungal signaling pathways, or indirectly via the alteration of the local microenvironment (e.g., pH, nutrient availability, redox state), thereby triggering adaptive transcriptional responses in the fungus. Specifically, genes governing virulence traits, stress tolerance, and metabolic adaptation are prime targets for such cross-kingdom regulation. Whether *H. pylori* influences *Candida* phenotype and virulence through these or other mechanisms remains largely unexplored. In this study, we therefore investigated whether co-culture with *H. pylori* alters the morphology and expression of virulence-associated genes and proteins in *Candida*.

## Materials and methods

### Strains, media, and cell culture

*H. pylori* (ATCC 700392, commonly referred to as strain 26695), *H. pylori* S7 (specifically used for experiments with murine models), *Candida albicans* (ATCC MYA-2876, commonly referred to as SC5314), and human gastric mucosal epithelial cells (GES-1) were used in this study. All strains were maintained at the Key Laboratory of Microbiology and Parasitology of the Education Department of Guizhou, Guizhou Medical University, Guiyang, China.

*H. pylori* strains were cultured in brain heart infusion (Oxoid, Basingstoke, UK) supplemented with 7% defibrinated sheep blood (Biological Industries, Henan, China) and incubated under microaerobic conditions at 37 °C for 48–72 h. *Candida* strains were cultured on Sabouraud dextrose agar (SDA) supplemented with 50 µg/mL chloramphenicol and incubated under aerobic conditions at 37 °C for 24 h. GES-1 cells were maintained in RPMI 1640 medium (Gibco, Thermo Fisher Scientific) supplemented with 10% fetal bovine serum (Tianhang, Hangzhou, China) at 37 °C in a humidified incubator with 5% CO_2_.

### Detection of H. pylori 16 S rRNA, ureA, and glmM genes in total DNA extracted from C. albicans

*H. pylori* 26,695 and *C. albicans* SC5314 were mixed at a ratio of 100:1, and the suspension was inoculated into yeast extract peptone dextrose (YPD; Basebio, Hangzhou, China) liquid medium. Cultures were incubated at 37 °C with shaking at 120 rpm for 24 h under microaerobic conditions. Amoxicillin (2 µg/mL) was added to the aforementioned culture medium, and the resulting mixture was incubated for 24 h to eliminate *H. pylori* present outside the *Candida* cells. The above-processed *C. albicans* was named CacoHp. Subsequently, 100 µL of *C. albicans* culture was inoculated onto SDA (Basebio, Hangzhou, China) supplemented with 50 µg/mL chloramphenicol (Solarbio, Beijing, China) and continuously passaged for five generations. The presence of *H. pylori*–specific genes (16 S rRNA, *ureA*, and *glmM*) in each passage of *C. albicans* was examined using previously described protocols [30–32]. DNA extracted from pure cultures of *H. pylori* 26,695 and *C. albicans* SC5314 served as positive and negative controls, respectively. Briefly, *Candida* cells were suspended in distilled water, and genomic DNA was extracted using a DAAN nucleic acid extraction or purification reagent (magnetic bead method; DAAN GENE, Guangdong, China). Nested PCR assays targeting 16 S rRNA, *ureA*, and *glmM* were performed to detect *H. pylori* genes in *C. albicans*. Primer sequences are listed in Table 1.

**Table 1:** Polymerase chain reaction primer sequences

PCR stage	Gene	Primers	Sequence	Amplimer size (bp)
First	16 S rRNA	HeliS	5′-AAGAACCTTACCTAGGCTTGACATTG-3′	497
		HeliN	5′-CCGTGGGCAGTAGCCAATT-3′	
Second		Hpup	5′-TGAGAGAATCCGCTAGAAATAGTGG-3′	454
		Hpdown	5′-TAGCATCCTGACTTAAGGCAAACA-3′	
First	*ureA*	PylF	5′-CCAGATGATGTGATGGATGG-3′	607
		PylR	5′-TCAAGTCTGTATCGCCCAATC-3′	
Second		HPU1	5′-GCCAATGGTAAATTAGTT-3′	411
		HPU2	5′-CTCCTTAATTGTTTTTAC-3′	
First	*glmM*	EHC-U	5′-CCCTCACGCCATCAGTCCCAAAAA-3′	417
		EHC-L	5′-AAGAAGTCAAAAACGCCCCAAAAC-3′	
Second		ET-5U	5′-GCCAAATCATAAGTCCGCAGAA-3′	230
		ET-5 L	5′-TGAGACTTTCCTAGAAGCGGTGTT-3′	

### Assessment of C. albicans responses to antifungal drugs, H_2_O_2_, and SDS

*C. albicans* was inoculated into YPD liquid medium at a final concentration of 2.5 × 10^3^ colony-forming units (CFU)/mL. Amphotericin B (AMB; 0.1, 0.05, and 0.025 µg/mL), fluconazole (FLU; 0.1, 0.05, and 0.025 µg/mL), caspofungin (CAS; 0.01, 0.005, and 0.0025 µg/mL), and H_2_O_2_ (20, 40, and 60 mM) were added to the cultures, followed by incubation at 37 °C with shaking at 150 rpm for 24 h. After incubation, cultures were serially diluted with normal saline (1:5, 1:25, 1:125, 1:625, and 1:3125), and 10 µL of each dilution was spotted onto YPD agar plates. Separately, a *C. albicans* suspension adjusted to 1 × 10⁶ CFU/mL was serially diluted (1:5, 1:25, 1:125,1:625, and 1:3125), and 5 µL of each dilution was plated onto YPD agar containing 0.02% SDS. Plates were incubated at 37 °C, and colony growth was assessed after incubation. All experiments were performed in triplicates.

### Cytotoxicity assay of C. albicans on GES-1 cells

SC5314 and fifth-passage CacoHp strains were counted and suspended in phosphate-buffered saline at a concentration of 1 × 10^6^ CFU/mL. The strains were inoculated into YPD liquid medium and incubated for 24 h. Cultures were then centrifuged and filtered through a 0.22-µm membrane filter. The resulting culture filtrates from SC5314 and CacoHp were diluted fivefold with RPMI 1640 medium and applied to GES-1 cells for 24 h. Cytotoxic effects were evaluated using a cell counting kit-8 (CCK) assay. The cell inhibition rate (%) was calculated as follows: [(absorbance value of control group – absorbance value of experimental group)/absorbance value of control group]×100%.

### Aspartic proteinase activity and hyphae formation assays of C. albicans

*C. albicans* cultures grown for 24 h were inoculated into YPD liquid medium and adjusted to a final concentration of 1 × 10^6^ CFU/mL. Concurrently, 10 µL of the *C. albicans* suspension was inoculated onto medium containing 0.2% bovine serum albumin (BSA). Cultures were incubated under aerobic conditions at 37 °C for 2 days, after which the diameter of the proteolytic halo was measured using a vernier caliper. Hyphal formation was assessed by observation under a microscope after incubation for 24 h at 37 °C on solid Spider medium and in YPD liquid medium supplemented with 10% fetal bovine serum, as previously described [33]. *Candida* hyphae on the Spider culture medium were quantified, and their length was measured under a microscope. The proportion of hyphal cells within 100 *Candida* cells in 10 µL of YPD liquid culture medium was determined. All experiments were performed in triplicates.

### Proteomic analysis of proteins of C. albicans

SC5314 and fifth-passage CacoHp strains cultured on SDA were rapidly frozen in liquid nitrogen and pulverized into a fine powder. The samples were homogenized in lysis buffer using ultrasonication (1 s on/1 s off cycles for 5 min). Following centrifugation at 14,000 × *g* for 20 min, the supernatant was collected, and protein concentration was determined using the Bradford assay. An appropriate amount of protein was supplemented with dithiothreitol to a final concentration of 5 mM and incubated at 37 °C for 1 h, followed by cooling to 25 °C. Iodoacetamide was then added to a final concentration of 10 mM, and samples were incubated in the dark at 25 °C for 45 min. The samples were diluted fourfold with 25 mM ammonium bicarbonate buffer, and trypsin was added at a protein-to-trypsin mass ratio of 50:1. Digestion was carried out overnight at 37 °C. The reaction was terminated by the addition of formic acid to adjust the pH to below 3.0. The resulting peptide mixtures were desalted using a C18 solid-phase extraction column. The column was sequentially conditioned with 100% acetonitrile and equilibrated with 0.1% formic acid. Samples were loaded, washed with 0.1% formic acid to remove impurities, and peptides were eluted with 70% acetonitrile. Eluates were collected and lyophilized for downstream analysis. Lyophilized peptides were analyzed by mass spectrometry using an Orbitrap Exploris™ 480 Mass Spectrometer (Thermo Fisher Scientific) equipped with an optional FAIMS Pro™ interface. Amino acid sequences were searched against a reference database constructed from the transcriptomic gene sequence file of *C. albicans* SC5314. Raw proteomic data were processed using Proteome Discoverer software. Quantitative protein values between groups were compared using Student’s *t*-test, and corresponding *p*-values were calculated. Multiple testing correction was performed using the Benjamini–Hochberg false discovery rate method.

### Quantitative PCR assay for the expression levels of the END3, SNQ2, ENO1, CTA1, and VMA8 genes in C. albicans

The relative expression levels of *END3*, *SNQ2*, *ENO1*, *CTA1*, and *VMA8* in *C. albicans* were assessed, with each sample analyzed in triplicates. First- and fifth-generation C*andida* strains were used for RNA extraction using the phenol–chloroform method. One microliter of total RNA was used as the template for complementary DNA synthesis using HiScript II Q RT SuperMix (+ gDNA wiper) (Vazyme Biotechnology Co., Ltd., Nanjing, China). Quantitative PCR (qPCR) was performed in a 96-well format on a CFX96 real-time PCR system (Bio-Rad, Hercules, CA, USA) using ChamQ qPCR SYBR Green Master Mix (Vazyme Biotechnology Co., Ltd.). *Candida ACT1* was used as the internal reference gene. Relative gene expression levels were calculated using the 2^-∆∆CT^ method. Primer sequences for *END3*,* SNQ2*,* ENO1*,* CTA1*, and *VMA8* are listed in Table 2.

**Table 2:** Primers used for quantitative PCR amplification

*SNQ2*	SNQ2 F	5′-TTTGACTGTTTGGCCACATG-3′
	SNQ2 R	5′-ACAACCTTCCACCGATGATG-3′
*END3*	END3 F	5′-CGTGGAGTGCCTGCTAGTTT-3′
	END3 R	5′-TGTACTACTCGCACCAACACC-3′
*ENO1*	ENO1 F	5′-AAACCCAGAATCCGACCCATC-3′
	ENO1 R	5′-GACCCAAGCATCCCAGTCATC-3′
*CTA1*	CTA1 F	5′-ACTCCAGTGTTTTTCATTAGAG-3′
	CTA1 R	5′-AGAGTAACCATTCATTTCTCTG-3′
*VMA8*	VMA8 F	5′-GCTCGATTCACCGTCAAAGC-3′
	VMA8 R	5′-CCACCTCTTGCTAAAGCCGT-3′
*ACT1*	ACT1F	5′-TTGGTGATGAAGCCCAATCC-3′
	ACT1R	5′-CATATCGTCCCAGTTGGAAACA-3′

### Establishment of a mouse model of C. albicans infection in the stomach

All animal experiments were approved by the Ethics Review Committee for Animal Experiments at Guizhou Medical University (approval number: 2200041). The mouse model of gastric infection was established in accordance with previously published studies [34]. Briefly, 4-week-old specific pathogen-free male BALB/c mice were obtained from Guizhou Huijiu Biotechnology Co., Ltd. and acclimatized for 1 week under controlled environmental conditions. Animals were housed at 22 ± 2 °C with a relative humidity of 50%±10% under a 12-h light/dark cycle. Mice were randomly assigned to four groups: control, SC5314, CacoHp (SC5314 + S7), and *H. pylori*(S7), with six mice per group. Infection was elicited by oral gavage with *C. albicans* or *H. pylori* suspensions standardized to 5 McFarland units. Gavage was performed every 48 h, and the mice were fasted for 12 h before each administration. One hour before inoculation, 0.3 mL of 0.1 M sodium bicarbonate solution was administered by gavage to neutralize gastric acidity. Each mouse then received 0.3 mL of the corresponding microbial suspension. The treatment regimen was continued for 8 weeks (28 administrations in total). The control group received an equivalent volume of normal saline. One week after the final gavage, blood samples were collected for serum separation, and serum levels of gastrin (GAS) and C-X-C motif chemokine ligand 1 (CXCL-1) were measured using enzyme-linked immunosorbent assay (ELISA) kits (Cusabio, Wuhan, China). Stomach tissues were excised, opened longitudinally, and divided into two equal portions. One portion was fixed in paraformaldehyde and processed for hematoxylin and eosin staining. The second portion was homogenized, and tumor necrosis factor-α (TNF-α) levels were quantified using an ELISA kit (Cusabio, Wuhan, China). Pathological scores were assigned based on the density of cells associated to chronic inflammation in the mucosal layer and in the depth of their infiltration. The researchers who performed the measurements were blinded to the experimental group each sample belonged to in order to avoid potential biases.

The extent of chronic inflammatory cell infiltration in the lamina propria was quantitatively scored. Specifically, 0 points signified no chronic inflammatory cell infiltration in the lamina propria; 1 point denoted a minor amount of chronic inflammatory cell infiltration in the lamina propria; 2 points represented a moderate amount of chronic inflammatory cell infiltration in the lamina propria; and 3 points indicated a substantial amount of chronic inflammatory cell infiltration in the lamina propria. Simultaneously, in accordance with the degree of gastric mucosal atrophy and intestinal metaplasia, they were respectively categorized and scored. Specifically, no atrophy or metaplasia was assigned 0 points, mild atrophy or metaplasia was assigned 1 point, moderate atrophy or metaplasia was assigned 2 points, and severe atrophy or metaplasia was assigned 3 points. Based on a comprehensive assessment of the above three indicators, scores were respectively allocated to the extent of chronic inflammatory cell infiltration in the lamina propria mucosa, the degree of gastric mucosal atrophy, and the degree of intestinal metaplasia, and the total score was calculated to comprehensively assess the condition of gastric mucosal lesions.

### Statistical analysis

Statistical analysis was performed using GraphPad Prism 9.5.0 software. All quantitative data are presented as mean ± standard deviation (SD) from at least three independent biological replicates. For comparisons between two groups, statistical significance was determined using a two-tailed unpaired Student’s t-test. For comparisons involving multiple groups, one-way analysis of variance (ANOVA) was used, followed by Tukey’s post-hoc test for multiple comparisons. For experiments with two independent variables, two-way ANOVA was performed, followed by Bonferroni’s post-hoc correction for multiple comparisons. A *p*-value < 0.05 was considered statistically significant.

## Results

### H. pylori 16 S rRNA, ureA, and glmM were detected in C. albicans

**Fig. 1:**
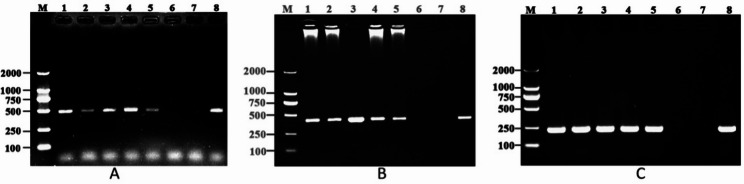
Detection of *Helicobacter pylori*-specific 16 S rRNA, *ureA*, and *glmM* within *Candida albicans*. *H. pylori*-specific 16 S rRNA, *ureA*, and *glmM* could be detected in each of the five consecutive generations of *C. albicans*. The amplification products of *H. pylori*-specific 16 S rRNA (**A**), *ureA* (**B**), and *glmM* (**C**) were separated by electrophoresis on a 1.5% agarose gel stained with GelRed and visualized under UV illumination. The DNA ladder, indicating base pair sizes, is displayed on the left side of the gel image. The *H. pylori*-specific 16 S rRNA (A), *ureA* (**B**), and *glmM* (**C**) amplified fragments were 454 base pairs, 411 base pairs, and 230 base pairs. Lanes 1–5: CacoHp strains from the first to fifth passages. Lane 6: negative control (*C. albicans* SC5314). Lane 7: blank control. Lane 8: positive control (*H. pylori* 26695). M: marker

Even after five consecutive passages, *H. pylori* 16 S rRNA, *ureA*, and *glmM* genes were detected in CacoHp by PCR (Fig. 1). These results indicate that co-culturing *H. pylori* with *C. albicans* can yield *C. albicans* strains that are positive for *H. pylori*–specific nucleic acids. In contrast, PCR assays for *H. pylori* 16 S rRNA, *ureA*, and *glmM* were negative in the SC5314 strain, confirming that parental *C. albicans* did not contain *H. pylori* nucleic acids. Because only PCR detection of *H. pylori*–related genes was performed, the quantity of *H. pylori* genetic material within *Candida* cells was not quantitatively determined. In addition, PCR results may be influenced by multiple technical factors, and no quantitative comparison was conducted among different *Candida* passages.

### H. pylori enhanced the tolerance of C. albicans to antifungal drugs, H_2_O_2_, and SDS

**Fig. 2:**
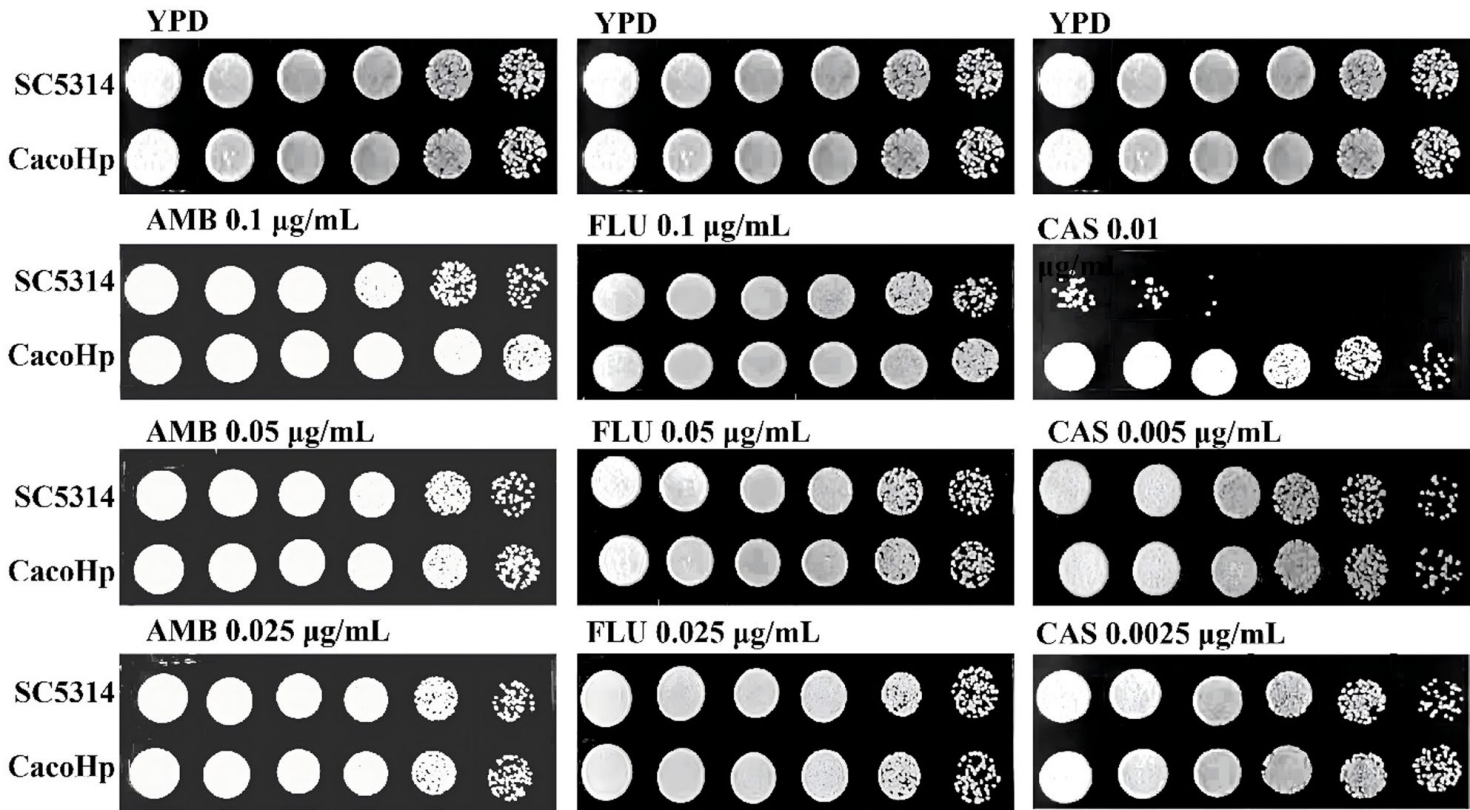
Detection of *Candida albicans* tolerance to AMB, FLU, and CAS. *C. albicans* was subcultured for five passages. The concentrations of AMB tested were 0.1, 0.05, and 0.025 µg/mL. The concentrations of FLU tested were 0.1, 0.05, and 0.025 µg/mL. The concentrations of CAS tested were 0.01, 0.005, and 0.0025 µg/mL. Results are representative of three independent experiments. The number of colonies of CacoHp was higher following exposure to 0.1 µg/mL AMB, 0.1 µg/mL FLU, and 0.01 µg/mL CAS. CacoHp exhibited significantly greater tolerance to these antifungal agents than standard *C. albicans* SC5314. AMB, amphotericin B; CAS, caspofungin; FLU, fluconazole

The high prevalence of *C. albicans* infections contributes substantially to the global burden of infectious disease–related mortality. Therapeutic options for these infections remain limited, and their effectiveness is further challenged by the increasing emergence of fungal strains exhibiting intrinsic or acquired tolerance to available antifungal agents. The presence of *H. pylori* may alter the composition or structure of the *C. albicans* cell wall and membrane, potentially affecting susceptibility to antifungal drugs that target these structures. To examine this possibility, we assessed the tolerance of *C. albicans* to three antifungal agents targeting the cell wall or membrane: AMB, FLU, and CAS. The CacoHp strain exhibited higher colony counts than the SC5314 strain at 0.1 µg/mL AMB, 0.1 µg/mL FLU, and 0.01 µg/mL CAS, indicating enhanced tolerance to these agents (Fig. 2).

**Fig. 3:**
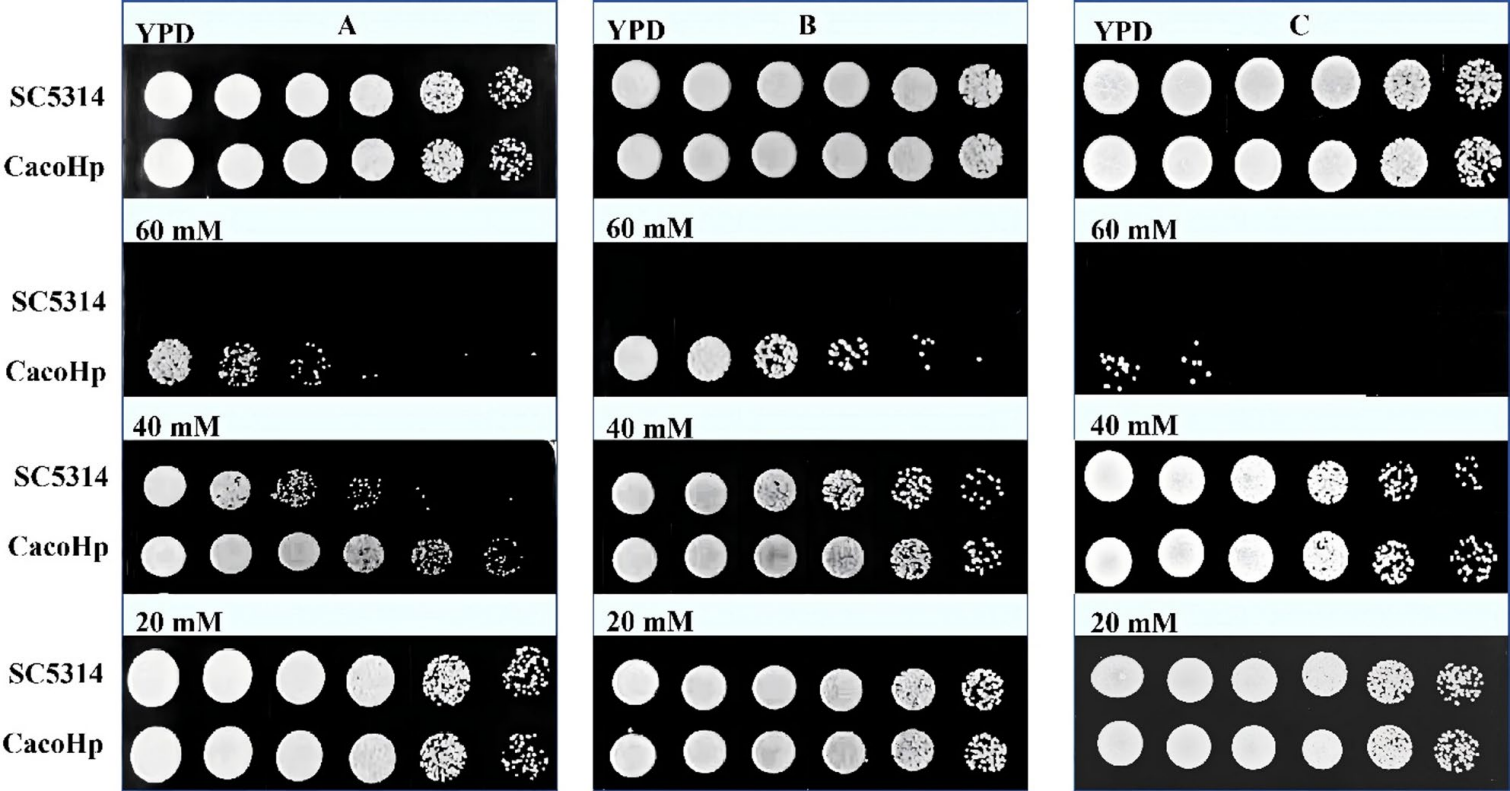
Differential sensitivity of *Candida albicans* cells to H_2_O_2_. (**A**) After 24 h co-culture of *C. albicans* SC5314 with *Helicobacter pylori* (Hp) 26,695. (**B**) CacoHp subcultured for one passage. (**C**) CacoHp subcultured for five passages. The concentrations of H_2_O_2_ used were 20, 40, and 60 mM. The results are representative of three independent experiments. Under treatment with 40 mM and 60 mM H_2_O_2_, colonies of CacoHp were still observed to grow, indicating enhanced tolerance to H_2_O_2_

**Fig. 4:**
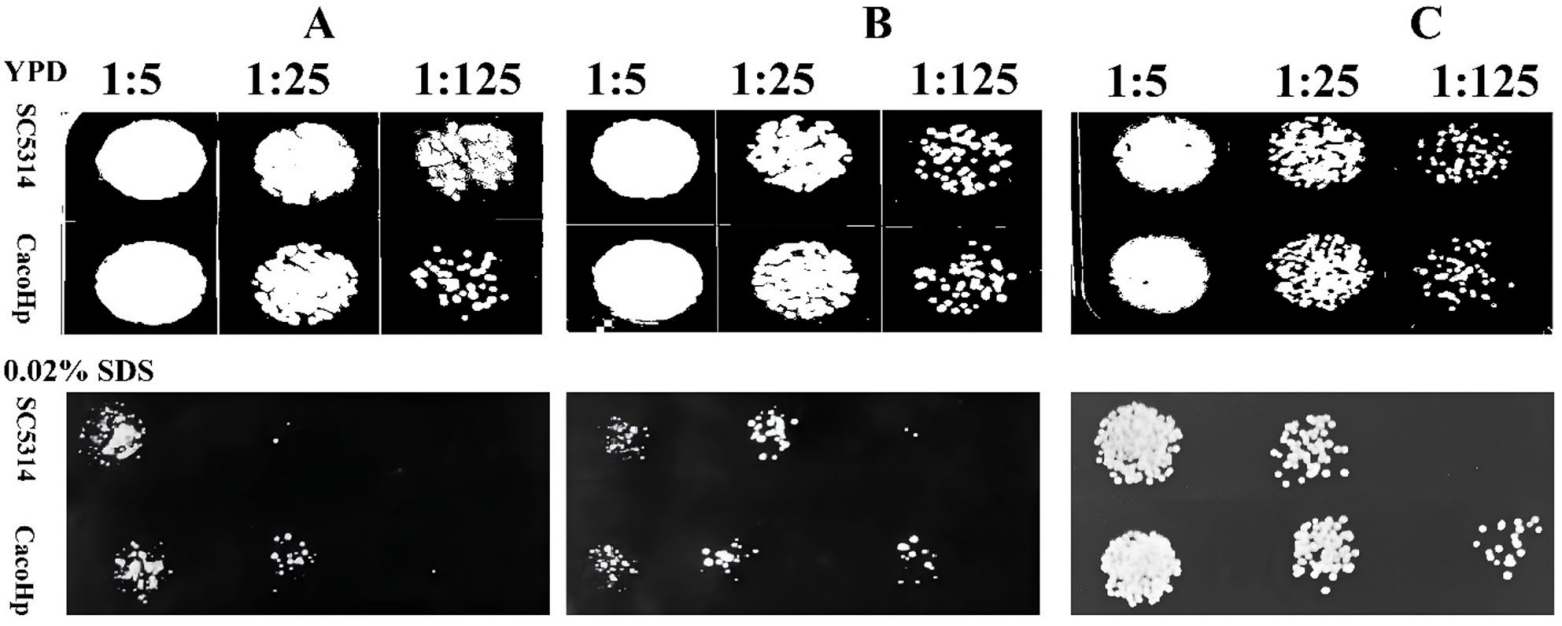
Detection of *Candida albicans* tolerance to SDS. The results are representative of three independent experiments. (**A**) After 24 h of co-culture of *C. albicans* SC5314 with *Helicobacter pylori* (Hp) 26,695. (**B**) CacoHp subcultured for one passage. (**C**) CacoHp subcultured for five passages. The CacoHp strain exhibited a higher colony count than the SC5314 strain at 1:25 and 1:125 dilutions. SDS, sodium dodecyl sulfate

SDS and H_2_O_2_ are commonly used cell surface-perturbing agents to assess membrane integrity and cellular stress tolerance. To further evaluate the effects of *H. pylori* on the physicochemical properties of *C. albicans*, tolerance to SDS and H_2_O_2_ was examined. Under SDS treatment, CacoHp displayed higher colony counts than SC5314 at 1:5 and 1:25 dilutions. Overall, CacoHp demonstrated enhanced tolerance to both SDS and H_2_O_2_ compared with the parental strain (Figs. 3 and 4). These findings suggest that association with *H. pylori* enhances the ability of *C. albicans* to withstand surface stress and adverse environmental conditions.

### CacoHp strain had a stronger inhibitory effect on the proliferation of GES-1 cells

**Fig. 5:**
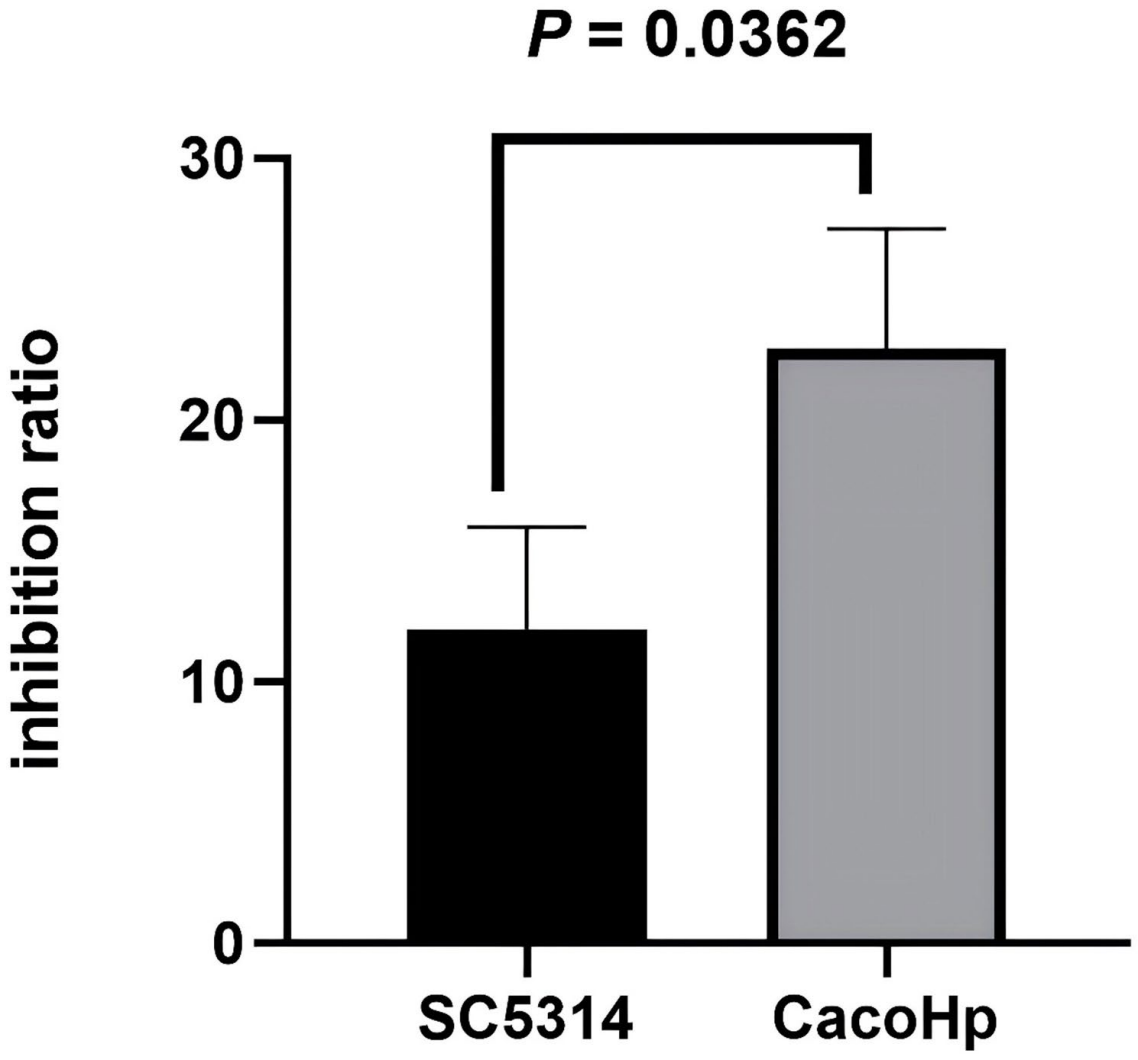
Inhibitory effect of *Candida albicans* on GES-1 cells. To investigate the alterations in virulence of *C. albicans*, we selected fifth-generation *C. albicans* and isolated its culture filtrate for analysis. The filtrate from the CacoHp strain demonstrated a significantly more potent inhibitory effect on GES-1 cells than that from the SC5314 strain

To evaluate changes in *C. albicans* virulence, GES-1 cells were treated with *C. albicans* culture filtrates, and growth-inhibitory effects were assessed using the CCK-8 assay. The results showed that filtrates from CacoHp strain exerted a significantly greater inhibitory effect on GES-1 cells proliferation than those from the SC5314 strain. These findings indicate that association with *H. pylori* enhances the inhibitory effect of *C. albicans* on GES-1 cells (Fig. 5).

### H. pylori enhanced the secretion of aspartic protease by C. albicans

**Fig. 6:**
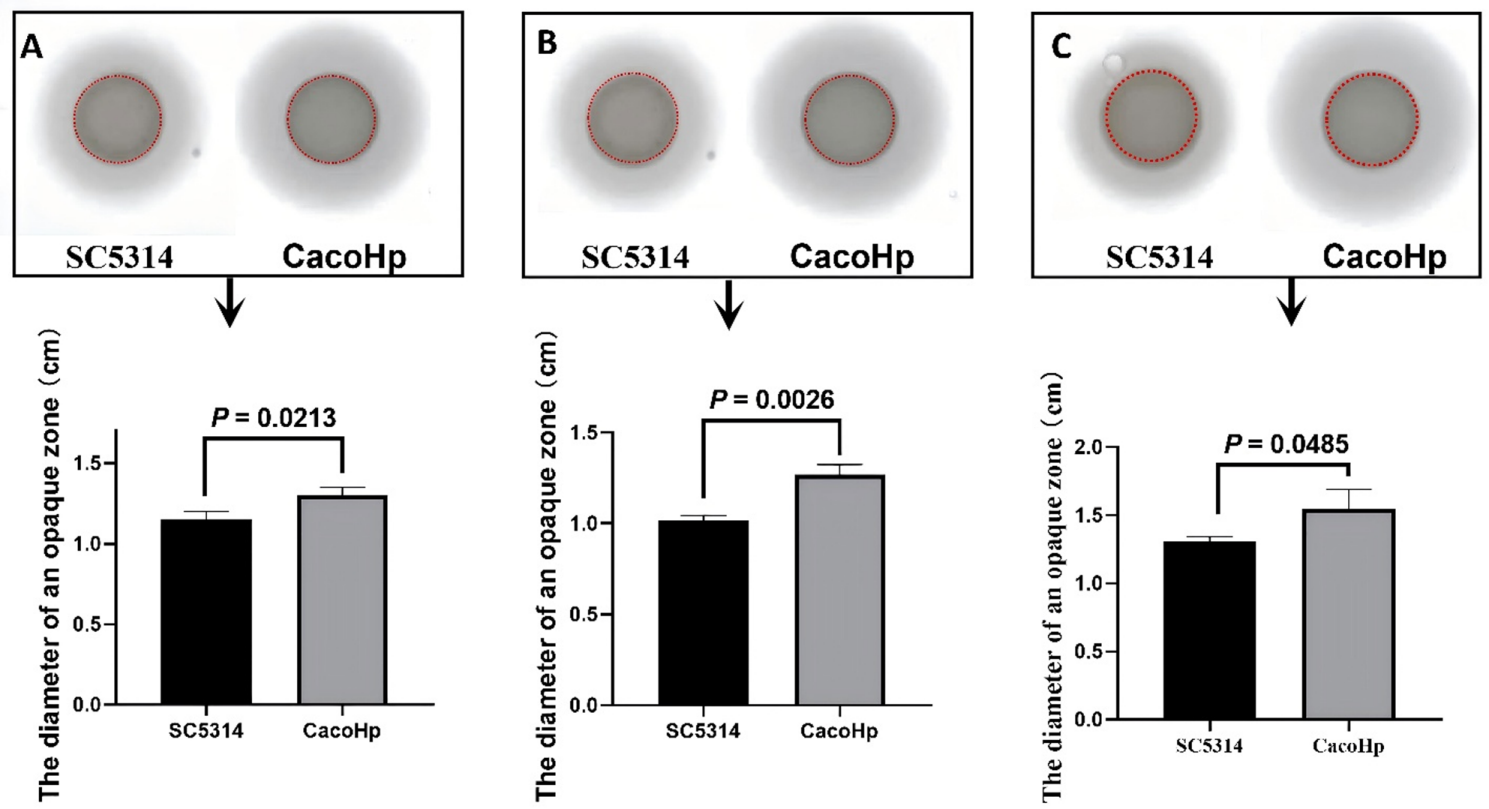
Diameter of the opaque zone. Results are representative of three independent experiments. (**A**) After 24 h co-culture of *Candida albicans* SC5314 with *Helicobacter pylori* (Hp) 26,695. (**B**) *C. albicans* subcultured for one passage. (**C**) *C. albicans* subcultured for five passages. The plaque size is indicated by the red circle, with the outer region depicting the halo formed owing to albumin breakdown mediated by aspartic proteinase. The plaque sizes of the SC5314 and CacoHp strains were consistent. The diameter of the halo reflects the extent of aspartic proteinase secretion. CacoHp had a larger halo, indicating that it had a stronger ability to secrete aspartic protease than SC5314

Aspartic proteinases are well-characterized secretory virulence factors of *C. albicans* [35] and play a pivotal role in fungal pathogenicity. To assess whether association with *H. pylori* affects protease secretion, aspartic proteinase activity was evaluated using BSA plate assays. The diameter of the opaque zone surrounding each colony was used as an indirect indicator of extracellular protease activity. Compared with the control strain SC5314, CacoHp strain exhibited a markedly enlarged zone of BSA degradation (Fig. 6). Similar results were observed when different generations of CacoHp were compared with SC5314. These findings indicate that CacoHp displays enhanced extracellular proteolytic activity, consistent with increased secretion of catalytically active aspartic proteinases.

### H. pylori enhances C. albicans hyphae formation

**Fig. 7:**
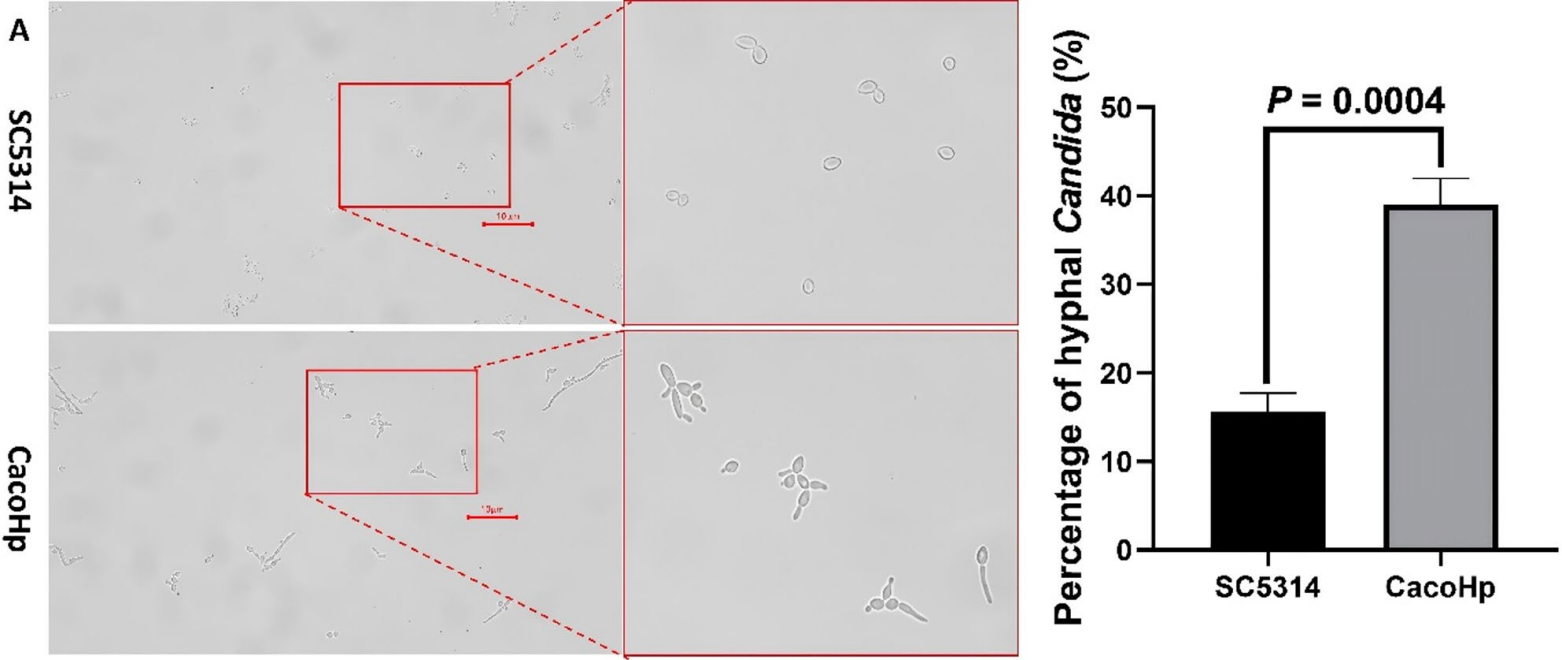

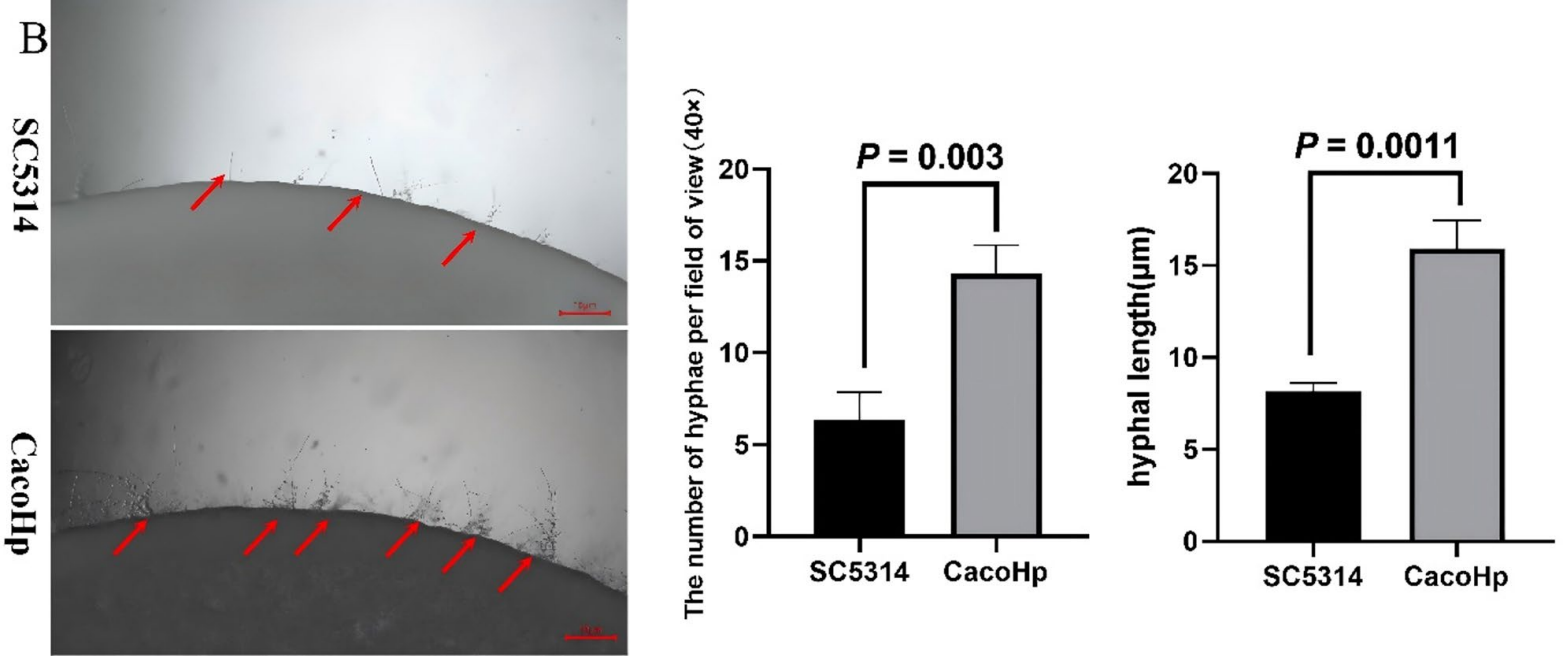

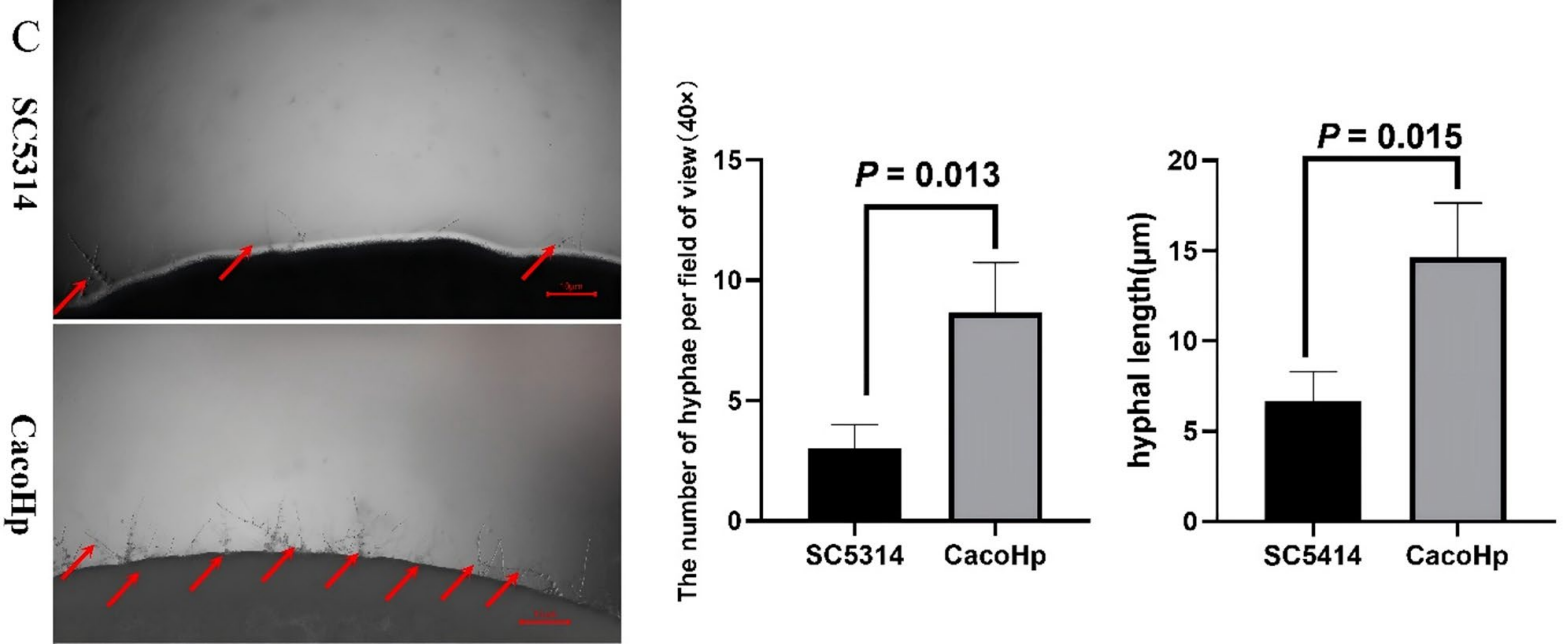
Hyphae formation. (**A**) Hyphal formation observed in liquid medium following a 24 h co-culture period of *Candida albicans* SC5314 with *Helicobacter pylori* (Hp) 26,695. (**B**) Hyphal formation observed in Spider medium. The upper panels correspond to *C. albicans* subcultured for one passage, (**C**) Hyphal formation observed in Spider medium. The lower ones to *C. albicans* subcultured for five passages. CacoHp strain has an enhanced ability to form hyphae. The red arrows indicate the budding cell bodies and hyphae

The transition from yeast to invasive hyphae is a key determinant of *C. albicans* virulence. Hyphal morphogenesis contributes to drug tolerance and plays a critical role during infection [36]. The morphological plasticity of *C. albicans* is influenced both by interactions with surrounding microbial communities and by environmental conditions [37]. Furthermore, virulence factors released by hyphal cells can promote fungal persistence and proliferation within the gut under conditions of increased bacterial competition [38]. To investigate the effect of *H. pylori* on *C. albicans* morphogenesis, hyphal formation was examined under inducing conditions. CacoHp strain exhibited a higher number of budding cell bodies (Fig. 7A) and produced more hyphae (Fig. 7B–C) than the SC5314 strain. The number of hyphae generated by CacoHp on Spider medium was higher than that of SC5314, and they had a higher length. Notably, enhanced hyphal production was consistently observed in CacoHp even after multiple subcultures. This sustained increase in hyphal formation is biologically relevant, as hyphae development is closely associated with *C. albicans* pathogenicity and virulence.

### H. pylori affects the proteomic expression and metabolism of C. albicans

**Fig. 8:**
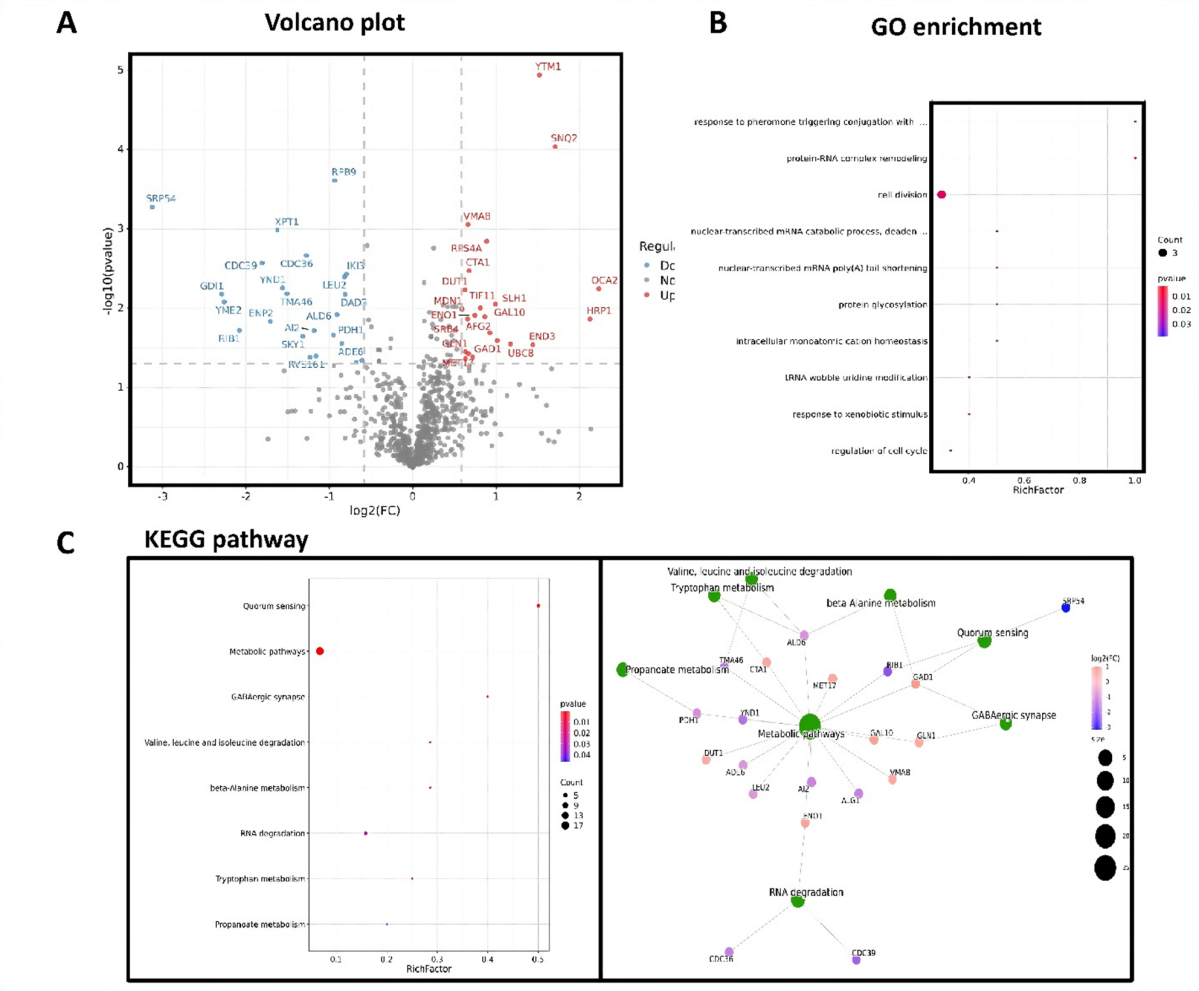
Proteomic analys. (**A**) Volcano plot of DEPs. (**B**) Gene ontology (GO) enrichment analysis of DEPs. (**C**) Kyoto Encyclopedia of Genes and Genomes (KEGG) pathway enrichment analysis of DEPs. *H. pylori* induces changes in the biosynthetic pathways and metabolic processes of *C. albicans*. These altered metabolic processes may promote changes in the virulence and tolerance of *C. albicans*, and enhance its adaptability to external stressors

To further investigate the mechanisms underlying the phenotypic changes observed in *C. albicans* following association with *H. pylori*, proteomic analyses were performed on SC5314 and CacoHp. Enzymatically digested peptides were analyzed by liquid chromatography-mass spectrometry. A total of 45 differentially expressed proteins (DEPs) were identified in CacoHp compared with the SC5314 strain, including 22 upregulated and 23 downregulated proteins (Table 3). Volcano plotsof the DEPs are shown in Figs. 8A.

**Table 3:** Differential protein expression in *Candida albicans*

Protein_IDs	Gene_ names	Protein_names	CacoHp/Ca	*P* value
			log2(FC)	
Q12019	MDN1	Midasin(Ribosome export/assembly protein 1)	0.5902	0.010317
P33317	DUT1	Deoxyuridine 5’-triphosphate nucleotidohydrolase	0.6235	0.005861
P32288	GLN1	Glutamine synthetase (GS)	0.6307	0.035424
P20604	SIT4	Serine/threonine-protein phosphatase PP1-1	0.6309	0.043014
P32569	SRB4	Mediator of RNA polymerase II transcription subunit 17	0.6565	0.013643
P32610	VMA8	V-type proton ATPase subunit D	0.6602	0.000879
P06106	MET17	Homocysteine/cysteine synthase	0.6648	0.037363
P15202	CTA1	Peroxisomal catalase A	0.6718	0.003368
P38911	FPR3	Peptidyl-prolyl cis-trans isomerase (PPIase)	0.7168	0.041302
P00924	ENO1	Enolase 1	0.7443	0.012306
P38912	TIF11	Eukaryotic translation initiation factor 1 A	0.8115	0.009892
P04397	GAL10	Bifunctional protein GAL10	0.865	0.01281
P0CX35	RPS4A	Small ribosomal subunit protein eS4A	0.8852	0.001436
P32794	AFG2	ATPase family gene 2 protein	0.9243	0.020457
P53327	SLH1	RQC trigger complex helicase SLH1	0.9912	0.008878
Q04792	GAD1	Glutamate decarboxylase (GAD)	1.0136	0.025682
P28263	UBC8	Ubiquitin-conjugating enzyme E2-24 kDa	1.1708	0.028435
P39013	END3	Actin cytoskeleton-regulatory complex protein END3	1.4394	0.028951
Q12024	YTM1	Ribosome biogenesis protein YTM1	1.5175	0.000011
P32568	SNQ2	Protein SNQ2	1.7055	0.000092
Q99383	HRP1	Nuclear polyadenylated RNA-binding protein 4	2.1253	0.013692
P53949	OCA2	Tyrosine-protein phosphatase-like protein OCA2	2.2322	0.005676
P20424	SRP54	Signal recognition particle subunit SRP54	-3.1273	0.000528
P39958	GDI1	Rab GDP-dissociation inhibitor (Rab GDI)	-2.2908	0.006668
P32843	YME2	Mitochondrial escape protein 2 (Protein RNA12)	-2.2662	0.00835
P38066	RIB1	GTP cyclohydrolase-2	-2.081	0.019065
P25655	CDC39	General negative regulator of transcription subunit 1	-1.8133	0.002691
P48234	ENP2	Ribosome biogenesis protein ENP2	-1.706	0.014692
P47165	XPT1	Xanthine phosphoribosyltransferase 1 (XPRT)	-1.6241	0.001039
P40009	YND1	Golgi apyrase	-1.5622	0.005559
Q12000	TMA46	Translation machinery-associated protein 46	-1.5096	0.006565
Q03656	SKY1	Serine/threonine-protein kinase SKY1 (SRPK)	-1.3219	0.022611
P06100	CDC36	General negative regulator of transcription subunit 2	-1.2789	0.002169
P16661	ALG1	Chitobiosyldiphosphodolichol beta-mannosyltransferase	-1.2353	0.041446
P03876	AI2	Putative COX1/OXI3 intron 2 protein	-1.1865	0.019078
P25343	RVS161	Reduced viability upon starvation protein 161	-1.1592	0.040227
Q12428	PDH1	Probable 2-methylcitrate dehydratase (2-MC dehydratase)	-0.9532	0.021761
P27999	RPB9	DNA-directed RNA polymerase II subunit RPB9	-0.9345	0.000246
P54115	ALD6	Magnesium-activated aldehyde dehydrogenase, cytosolic	-0.9132	0.012043
P38972	ADE6	Phosphoribosylformylglycinamidine synthase	-0.8519	0.027856
P04173	LEU2	3-isopropylmalate dehydrogenase	-0.8237	0.004031
P69850	DAD3	DASH complex subunit DAD3	-0.815	0.006686
Q06706	IKI3	Elongator complex protein 1	-0.7949	0.003718
P33307	CSE1	Importin alpha re-exporter	-0.6771	0.048261
Q99297	ODC2	Mitochondrial 2-oxodicarboxylate carrier 2	-0.6099	0.0456

Subsequently, Gene Ontology and Kyoto Encyclopedia of Genes and Genomes enrichment analyses were conducted for the upregulated and downregulated DEPs. The analyses revealed that the DEPs were closely associated with biosynthetic pathways (Fig. 8B) and metabolic processes in *C. albicans* (Fig. 8C). GO enrichment indicated alterations in molecular function, cellular components, and multiple biosynthetic and metabolic processes, suggesting that with *H. pylori* induced significant changes *C. albicans* metabolic pathways. Notably, affected processes included biosynthesis, ribosomal small subunit biogenesis, double-strand break repair, DNA unwinding during replication, DNA replication initiation, and DNA recombination. These metabolic and biosynthetic alterations may contribute to the enhanced resilience of *C. albicans* to external stressors.

### Relative expression of C. albicans genes detected via qPCR

**Fig. 9:**
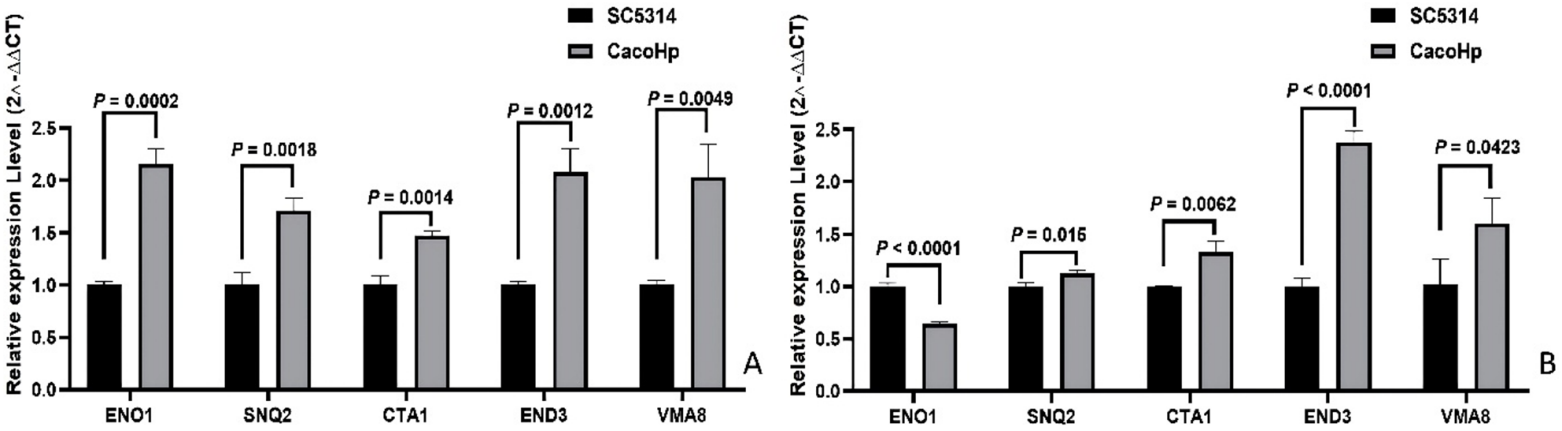
Relative expression of *Candida albicans* genes. (**A**) *C. albicans* subcultured for one passage. (**B**) *C. albicans* subcultured for five passages. CacoHp exhibited higher expression levels of *END3*, *SNQ2*, *ENO1*, *VMA8*, and *CTA1* than *C. albicans* SC5314 in first-passage. CacoHp exhibited higher expression levels of *END3*, *SNQ2*, *VMA8*, and *CTA1* than *C. albicans* SC5314 in fifth-passage

Based on the proteomic findings, several proteins associated with *C. albicans* virulence and tolerance were upregulated. Previous proteomic analyses indicated significant differences in the expression of *END3*, *SNQ2*, *ENO1*, *CTA1*, and *VMA8*, genes known to be involved in hyphal formation, virulence, and drug tolerance. To validate these findings at the transcriptional level, qPCR was performed. The results demonstrated that the first passage CacoHp exhibited higher expression levels of *END3*, *SNQ2*, *ENO1*, *VMA8*, and *CTA1* compared with the SC5314 strain (Fig. 9). The *ENO1* gene showed an opposite trend of change as the number of passages increased. These transcriptional changes are consistent with the proteomic data and further support the involvement of *H. pylori* in modulating *C. albicans* hyphal development, virulence, and tolerance to stress.

### H. pylori enhances the pathogenic ability of C. albicans in the stomach

**Fig. 10:**
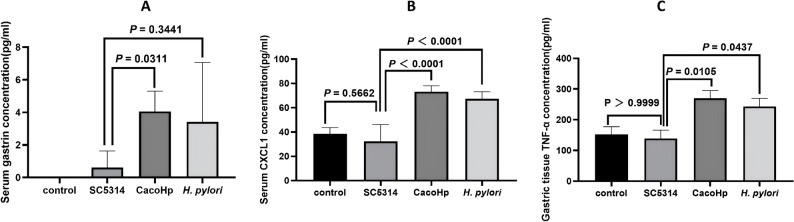
Inflammatory factor detection. (**A**) Detection of gastrin in mouse serum. (**B**) Detection of CXCL-1 in mouse serum. (**C**) Detection of TNF-α in mouse gastric tissue. CacoHp group mice exhibited higher expression levels of gastrin, CXCL-1, and TNF-α than *C. albicans* SC5314 group mice. Six mice were included in each group.

**Fig. 11:**
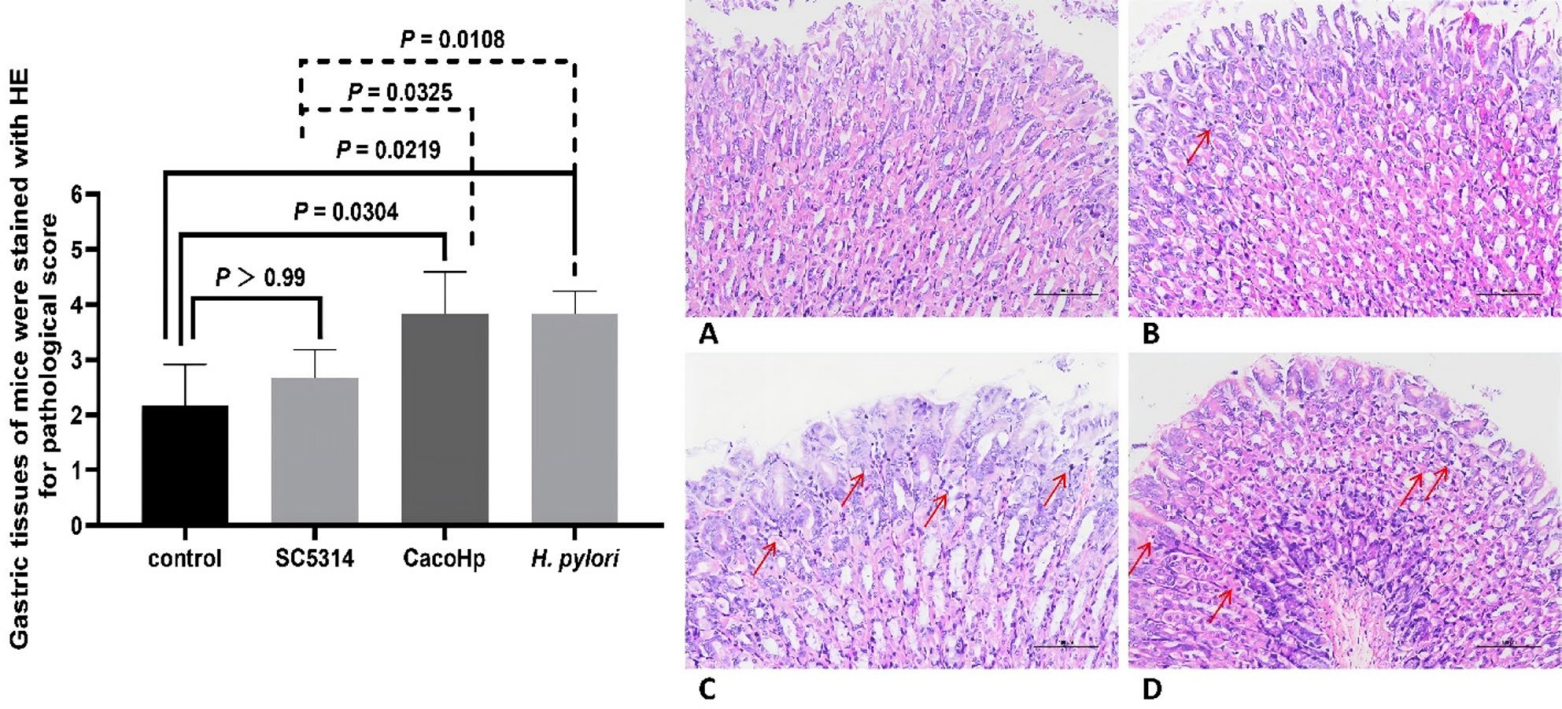
H&E staining of the gastric mucosa. (**A**) Control group. (**B**) SC5314 group. (**C**) CacoHp group. (**D**) *H. pylori* group. The inflammatory cell infiltration and bleeding in the CacoHp group were more obvious, and the inflammatory response was stronger than SC5314. The red arrow indicates the inflammatory cells. Six mice were included in each group. Magnification: ×200. H&E, hematoxylin and eosin

In mice infected with CacoHp, serum GAS and CXCL-1 levels were increased, and TNF-α levels in gastric tissue were elevated (Fig. 10A–C). These findings indicate that the presence of CacoHp enhances the inflammatory response in vivo. Histopathological examination using H&E staining further revealed increased infiltration of inflammatory cells in the gastric tissues of mice in the CacoHp group (Fig. 11). Correspondingly, pathological scores were higher in this group than in the SC5314 group. Together, these results suggested that CacoHp promotes gastric inflammation and exhibits greater pathogenicity in the stomach compared with the parental strain.

## Discussion

In this study, by utilizing the co-culture method, it was demonstrated that the specific nucleic acids (16 S rRNA, *ureA*, *glmM*) of *H. pylori* can persist within the cells of *C. albicans*. Furthermore, this HP-positive *C. albicans* has experienced substantial alterations in multiple virulence-related phenotypes. These alterations encompass enhanced tolerance to antifungal drugs and environmental stress, improved protease secretion ability, promoted mycelial morphological transformation, modified proteomic profiles, and ultimately, increased pathogenicity as manifested in in vivo models. Our findings offer novel experimental evidence for comprehending the intricate microbial interactions within the stomach, particularly how bacteria - fungal cross - border interactions influence pathogen virulence and disease progression.

The interaction between bacteria and fungi represents a highly intricate phenomenon within microbial ecology. These microorganisms establish intimate physical associations in diverse environments, encompassing mixed biofilms, ectosymbiosis, and endosymbiosis, among other varieties [1]. At the molecular level, their interactions are extensively and precisely regulated, and they communicate via mechanisms such as antibiotic action, signal transduction, regulation of the physicochemical environment, chemotaxis and cell contact, nutrient interaction, and cooperative metabolism, as well as protein secretion and gene transfer [39]. These interactions significantly impact the growth and development, pathogenicity, physiological state, survival adaptation, and dissemination of fungi [40]. In clinical contexts, the interactions between bacteria and fungi also exert dual effects: They can synergistically exacerbate the infection process, facilitate biofilm formation, and enhance drug resistance [41], or inhibit the growth of pathogenic bacteria through competition or antagonism, thereby offering novel perspectives for the prevention and treatment of infections [42].

Fungal infections are a growing global health concern and pose a significant threat to human health. Approximately 1.7 billion people worldwide experience fungal infections annually, underscoring their widespread impact [43]. Among the causative agents of nosocomial fungal infections, *Candida* species are the most prevalent [44]. *Candida* is highly adaptable due to its capacity to survive and proliferate across diverse host environments [45]. As a commensal organism, *Candida* occupies an ecological niche within the human microbiome, where it interacts with a broad range of microorganisms. In vivo, *Candida* frequently encounters co-infecting pathogens, and interactions between *Candida* and *H. pylori* have increasingly attracted attention.

*H. pylori* colonizes the gastric mucosa and is a major etiological agent of gastric ulcers, gastric lymphoma, and gastric cancer [46]. Fungal colonization of the gastric mucosa has been reported in 30%–50% of patients with active ulcer disease, where it may prolong symptom persistence and delay ulcer healing. Co-infection with *H. pylori* and *Candida* has been documented in patients with large gastric ulcers, and combined antifungal and *H. pylori* eradication therapy has been shown to shorten treatment duration and accelerate ulcer healing, suggesting a pathogenic association between ulcers and fungal infection [47]. Moreover, the coexistence of fungi and *H. pylori* may exert synergistic effects on ulcer pathogenesis [48]. Despite these observations, the mechanisms underlying *Candida*–*H. pylori* interactions remain incompletely understood.

In the present study, co-culture of *H. pylori* with *C. albicans* generated *C. albicans* strains positive for *H. pylori*–specific genes, which remained detectable after multiple passages. Consistent with our previous findings, *H. pylori* nucleic acids and antigens have been identified in *Candida* isolates from vaginal secretions and fecal samples, supporting a potential symbiotic or interdependent relationship between *H. pylori* and *Candida in vivo* [26]. Accumulating evidence suggests that *H. pylori* may be internalized within *Candida*, potentially using intracellular vesicles as protective niches to withstand unfavorable conditions [49–52]. The persistence of *H. pylori* nucleic acids within *C. albicans* during serial passages in vitro aligns with this proposed intracellular association.

Antifungal drug tolerance represents an increasing clinical challenge, particularly given the limited number of available antifungal drug classes. Notably, tolerance has been reported even in patients without prior antifungal exposure [53]. In this study, CacoHp exhibited increased tolerance to AMB, FLU, and CAS. These findings suggest that association with *H. pylori* modulates the antifungal tolerance profile of *C. albicans*. Importantly, this enhanced tolerance was not driven by direct antifungal exposure, offering a novel perspective on the mechanisms underlying antifungal tolerance. Increased tolerance to SDS and H_2_O_2_ further indicates alterations in cell wall structure and composition, potentially enhancing resistance to environmental stressors. These tolerance-associated changes persisted across multiple passages, suggesting a stable phenotypic alteration. Antifungal tolerance and environmental resilience are recognized virulence-associated traits, particularly in hospital settings where opportunistic pathogens can contribute to substantial morbidity and mortality [54, 55]. These findings also partially account for the natural drug resistance observed in certain clinical *Candida* isolates.

In parallel, *C. albicans* exhibited notable phenotypic changes during interaction with *H. pylori*. Hyphal formation was enhanced in CacoHp. Morphological dimorphism is a critical determinant of *C. albicans* virulence, influencing immune evasion and tissue invasion. While yeast forms facilitate dissemination, filamentous forms promote invasion and pathogenicity in disseminated candidiasis [56]. CacoHp also exerted stronger inhibitory effects on GES-1 cells and demonstrated increased secretion of aspartic proteases. Aspartic proteases are key virulence factors that facilitate host tissue invasion by degrading mucosal barriers and immune defense proteins [57]. Enhanced hyphal formation accompanied by increased protease secretion likely contributes to the elevated pathogenic potential observed in CacoHp.

Alterations in tolerance and virulence may be driven by *H. pylori*–induced reprogramming of *C. albicans* gene expression. Our study demonstrates that CacoHp exhibits enhanced tolerance to chemical stressors and increased virulence. The molecular basis for these phenotypic shifts likely involves a multi-layered regulatory network influenced by the presence of *H. pylori*. Proteomic and qPCR analyses revealed upregulation of key genes associated with stress response and pathogenicity in CacoHp. For instance, the elevated expression of SNQ2, an ABC transporter, may contribute to antifungal tolerance by regulating ergosterol trafficking to the cell membrane, thereby reducing drug permeability and enhancing membrane stability under azole pressure. The increased levels of *ENO1* and *END3* are closely linked to hyphal morphogenesis, cell wall integrity, and extracellular protease secretion, providing a structural basis for the enhanced invasive capacity observed in CacoHp strains. Furthermore, upregulation of *CTA1*, encoding a catalase, may improve fungal survival in oxidative stress environments by degrading host-derived hydrogen peroxide, while *VMA8*, a vacuolar proton ATPase subunit, could modulate intracellular pH and ion homeostasis, potentially creating a favorable niche for *H. pylori* persistence within fungal cells and augmenting *C. albicans* adaptability to nutritional stress. Collectively, these findings indicate that *H. pylori* alters *C. albicans* metabolism and enhances its capacity to withstand environmental stress. Given the complexity of *Candida* gene regulation, further validation using approaches such as RNA sequencing and gene knockout studies will be necessary to elucidate the precise molecular mechanisms involved.

Additionally, proteomic data indicated significant alterations in proteins involved in biosynthesis, metabolic pathways, and DNA repair in CacoHp. This suggests that *H. pylori* may reconfigure the metabolic network of *C. albicans*, redirecting energy resources and stress response strategies to maintain growth advantage under competitive or symbiotic conditions. Such metabolic reprogramming could represent an adaptive strategy evolved by the fungus to sustain or even enhance its pathogenic potential during cohabitation with bacteria.

Consistent with the in vitro findings, the murine model experiments revealed increased levels of inflammatory mediators, including GAS, CXCL-1, and TNF-α, in mice infected with CacoHp. Histopathological analysis further demonstrated more severe gastric inflammation in this group compared with mice infected with the SC5314 strain. These results suggest that *H. pylori* increases the pathogenic effects of *C. albicans in vivo* and exacerbates gastric inflammatory responses. A previous study by Heingrach et al. [34] demonstrated that *Candida* can serve as a reservoir for *H. pylori*, and that co-infection with both microorganisms results in exacerbated gastric inflammation compared to infection with either pathogen alone. These findings established an important foundation for understanding the synergistic relationship between these two organisms. Our study extends these observations by observing the influence of *H. pylori* on the phenotypes of *Candida*. While Heingrach et al. focused on histopathological changes and bacterial colonization, our work provides complementary insights into the phenotypic alteration of *Candida*. Taken together, these studies collectively advance our understanding of polymicrobial interactions in the gastric mucosa and their contribution to gastritis pathogenesis. However, *Candida* may have served as a carrier for *H. pylori*. Therefore, the inflammatory changes in mice might be caused by the *H. pylori* released by *Candida*. The pathogenicity impact of these phenotypic changes of *Candida* in mice still requires further experimental research.

While this study provides evidence that *H. pylori* presence alters *C. albicans* phenotype and virulence, several limitations should be noted. The regulatory mechanisms—such as *H. pylori*-derived signals or metabolic interplay—remain unknown. Transcriptomic, whole genome sequencing or dual-RNA sequencing could be used in the future to elucidate these inter-kingdom interactions. Additional clinical research is required to validate the clinical manifestations of their interaction. Longer co-evolution studies are also needed to reveal adaptive genetic changes influencing pathogenicity.

## Conclusions

In summary, our findings demonstrate that CacoHp exhibits enhanced tolerance to environmental stressors, altered phenotypic characteristics, and increased virulence. These changes may be accompanied by modifications in cell wall properties, gene expression, and metabolic pathways. The interaction between *C. albicans* and *H. pylori* may therefore create conditions that facilitate synergistic pathogenicity by both microorganisms, with potential implications for gastric disease progression.

## Data Availability

All data generated or analyzed during this study are included in the published article and its supplementary information files.

## Abbreviations

AMB: Amphotericin B
ATP: Adenosine triphosphate
BSA: Bovine serum albumin
CAS: Caspofungin
CFU: Colony-forming units
DEPs: Differentially expressed proteins
DNA: Deoxyribonucleic acid
ELISA: Enzyme-linked immunosorbent assay
END3: Actin cytoskeleton-regulatory complex protein
ENO1: Enolase 1
FLU: Fluconazole
GAS: Gastrin
GES-1: Human gastric epithelial cell line
glmM: Phosphoglucosamine mutase gene of Helicobacter pylori
Hp: Helicobacter pylori
H_2_O_2_: Hydrogen peroxide
CXCL-1: C-X-C motif chemokine ligand 1
PCR: Polymerase chain reaction
qPCR: Quantitative real-time polymerase chain reaction
RNA: Ribonucleic acid
rRNA: Ribosomal RNA
SDA: Sabouraud dextrose agar
SDS: Sodium dodecyl sulfate
SNQ2: Multidrug resistance ABC transporter SNQ2
TNF-α: Tumor necrosis factor alpha
VMA8: V-type proton ATPase subunit D
YPD: Yeast extract peptone dextrose
